# Cardiovascular magnetic resonance assessment of left atrial volumes and function in patients with persistent atrial fibrillation before and after ablation therapy

**DOI:** 10.1186/1532-429X-15-S1-E98

**Published:** 2013-01-30

**Authors:** Lay Koon Tan, Shouvik Haldar, Rick Wage, Jennifer Keegan, Tom Wong, Raad H Mohiaddin

**Affiliations:** 1 CMR, Royal Brompton Hospital, London, UK; 2 National Heart & Lung Institute, Imperial College, London, UK; 3 Cardiology, National Heart Institute, Kuala Lumpur, Malaysia

## Background

Atrial fibrillation (AF) disrupts coordinated atrial electrical and mechanical function which may lead to adverse atrial remodelling if persistent. This study aims to assess both left atrial (LA) volume and function by cardiovascular magnetic resonance (CMR) in patients with persistent AF both before and after restoration to sinus rhythm by ablation therapy.

## Methods

A Siemens scanner (Avanto, 1.5T) was used to study 13 patients (mean age 65 ± 9, 4 females and 9 males) with persistent AF before and 3 months after ablation therapy. Images of the LA were acquired in the two-chamber and four-chamber orientation using a breath-hold ECG-gated steady state free precession cine sequence. Biplane area-length method was used to measure the LA end-systolic (ESV) and end-diastolic (EDV) volumes[1]. LA function was determined by its calculated ejection fraction (EF=(EDV-ESV)/EDV) [1]. Seven-day rhythm tape was used to assess patients' rhythm 3 months after ablation. All 13 patients underwent either surgical or catheter ablation therapy successfully, achieving sinus rhythm post procedure. Only 2 patients reverted to AF at 3 months.

## Results

There is a significant reduction in both indexed (volumes by body surface area) LA ESV and EDV 3 months after ablation therapy (iESV baseline 51±13.8 ml, 3 months 36.3±13.1 ml, p=0.01; iEDV baseline 59.2±14.8 ml, 3 months 48.6±13.4 ml, p=0.03). LA EF showed a marked improvement from a baseline of 14.8±6.1% to 26.8±10.8% at 3 months, p=0.006. Of the 2 patients in sinus rhythm post ablation but reverted to AF at 3 months, the trend is similar (iESV 67.2±0.4 ml baseline, 52±15.9 ml at 3 months; iEDV 74.6±6.2 ml baseline, 64.7±5.4 ml at 3 months; Baseline EF 9.6±7%, 3 months 20.4±17.9%).

## Conclusions

We have demonstrated a marked reduction in CMR measured LA volumes and improvement in LA EF in patients with persistent AF three months after restoration to sinus rhythm by ablation therapy. This favourable change appears to be sustained even in patients with only transient sinus rhythm.

## Funding

No Funding.
